# ECMO combined with sequential oxygen therapy in drowning-induced ARDS: a case report

**DOI:** 10.3389/fmed.2025.1609610

**Published:** 2025-06-11

**Authors:** Yaxu Wu, Rui Zhai, Boyuan Wang, Pengpeng Zhao, Lijun Zhao, Huanhuan Cui, Chen Chen, Mei Hu

**Affiliations:** Department of Critical Care Medicine, The Ninth Medical Center of Chinese People’s Liberation Army General Hospital, Beijing, China

**Keywords:** drowning, extracorporeal membrane oxygenation, sequential oxygen therapy, acute respiratory distress syndrome, case report

## Abstract

A 35-year-old male was admitted with drowning-induced cardiorespiratory arrest. Initial assessment revealed acute respiratory distress syndrome (ARDS), pneumothorax and refractory hypoxemia. Despite mechanical ventilation and thoracostomy tube placement, his condition deteriorate. Prompting urgent initiating of veno-veous extracorporeal membrane oxygenation (V-V ECMO). Respiratory support was dynamically adjusted through sequential oxygen therapy, balancing oxygenation optimization with mitigation of ventilator-induced lung injury. Concurrent targeted antimicrobial therapy and intensive care management led to gradual clinical improvement, culminating in successful ECMO weaning and eventual recovery. This case highlights the potential of integrating ECMO with sequential oxygen therapy to address complex pathophysiological challenges in drowning-associated ARDS.

## Introduction

Drowning, the third leading cause of unintentional injury-related deaths globally, poses significant challenges in emergency and critical care management (1). With an estimated 300,000 annual fatalities worldwide (WHO data) its pathophysiological complexity-characterized by hypoxic injury, non-cardiogenic pulmonary edema, multiorgan dysfunction—demands therapeutic strategies that reconcile rapid intervention with physiological precision (2, 3). Current guidelines emphasize the *drowning survival chain*, spanning prevention, prehospital resuscitation (e.g., prompt retrieval from water, high-quality CPR, airway clearance) (4); and hospital-based management targeting hypoxemia correction (via high-flow oxygen or mechanical ventilation), hemodynamic stabilization, and multiorgan monitoring (5). For refractory hypoxemia unresponsive to conventional therapies, extracorporeal membrane oxygenation (ECMO) may serve as a salvage intervention (6).

Drowning-induced lung injury arises from alveolar-capillary membrane disruption and pulmonary surfactant loss, precipitating acute respiratory distress syndrome (ARDS) (7). While the EOLIA trial subgroup analysis supports early ECMO initiation in severe ARDS to improve outcomes (8), recent retrospective data suggest lower survival rates in drowning patients receiving ECMO than previously reported, with uncertain mortality impacts of distinct ECMO modalities (9). Despite these insights, clinical decision-making remains constrained by limited high-quality evidence, as most drowning-related studies are observational and fail to address critical knowledge gaps (10), including optimal ECMO timing, configuration, and adjuvant therapies. Impact of ECMO treatment on resuscitation of drowning victims needs further research to assess it (11).

This case report details the successful management of drowning-induced ARDS through ECMO combined with sequential oxygen therapy—a strategy dynamically balancing oxygenation enhancement and ventilator-induced lung injury mitigation. Our approach may provide a clinical reference for this high-risk population.

## Case report

Following retrieval from a 5-meter-deep swimming pool where the patient was found unresponsive with cardiopulmonary arrest (CPA), prompt CPR was initiated. During prehospital transport, oxygenation was sustained via a bag-valve-mask (BVM) device, accompanied by continuous norepinephrine infusion (0.4 μg/kg/min) to maintain hemodynamic stability.

**Figure 1:**
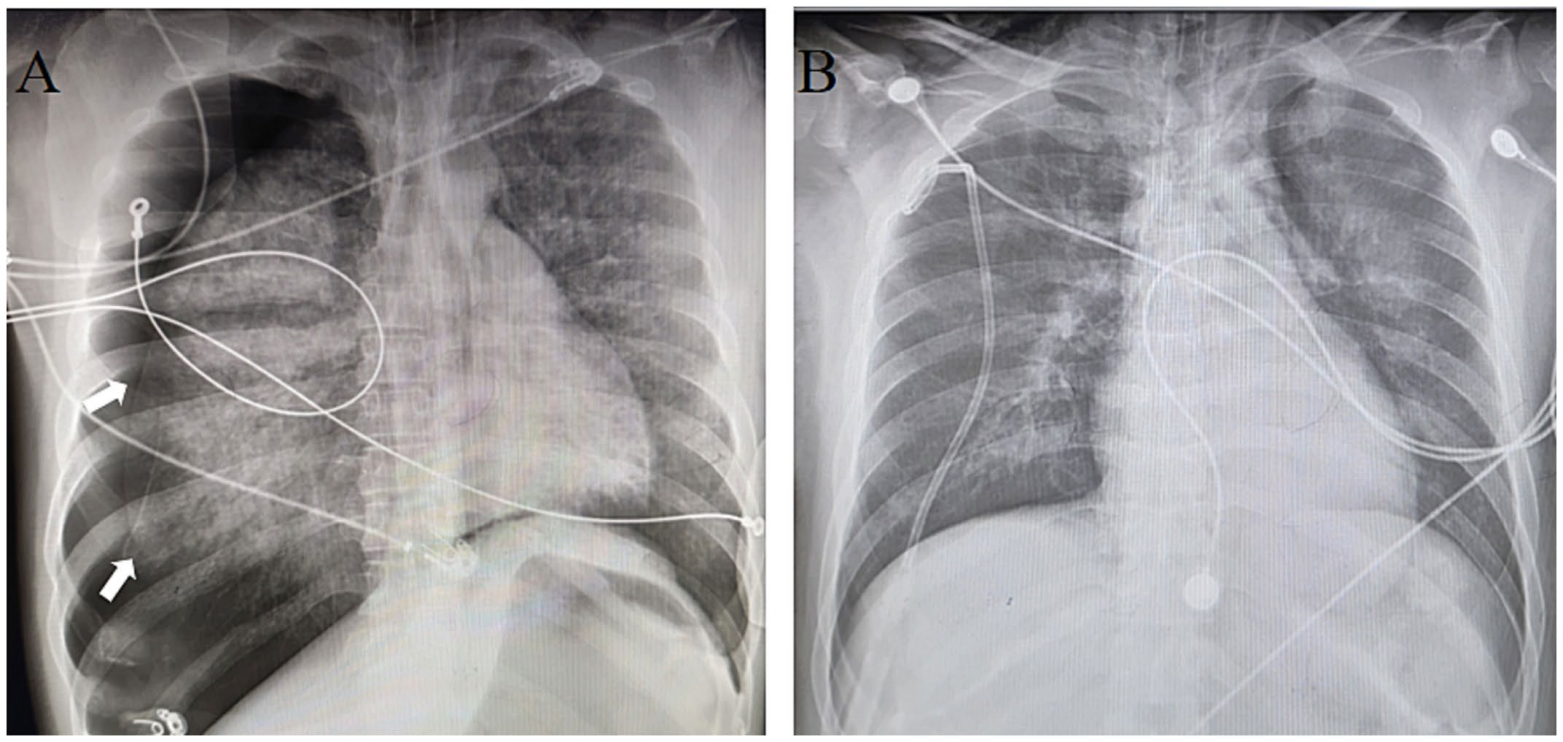
Chest X-ray. **(A)** Pneumothorax, white arrows: absence of lung markings. **(B)** Marked resolution of pneumothorax after thoracostomy tube placement.

Upon emergency department arrival, the patient presented comatose with perioral cyanosis, bilaterally diminished breath sounds, and SpO₂ 80% despite maximal oxygen supplementation. Emergent endotracheal intubation with mechanical ventilation was initiated. However, clinical deterioration ensued: chest X-ray demonstrated right-sided pneumothorax (>50% lung collapse, Figure 1A), necessitating immediate thoracostomy tube placement. The patient developed refractory hypotension requiring norepinephrine titration (0.2 μg/kg/min) and was subsequently transferred to the intensive care unit (ICU) for advanced hemodynamic monitoring and organ support.

### Admission examination

**Figure 2:**
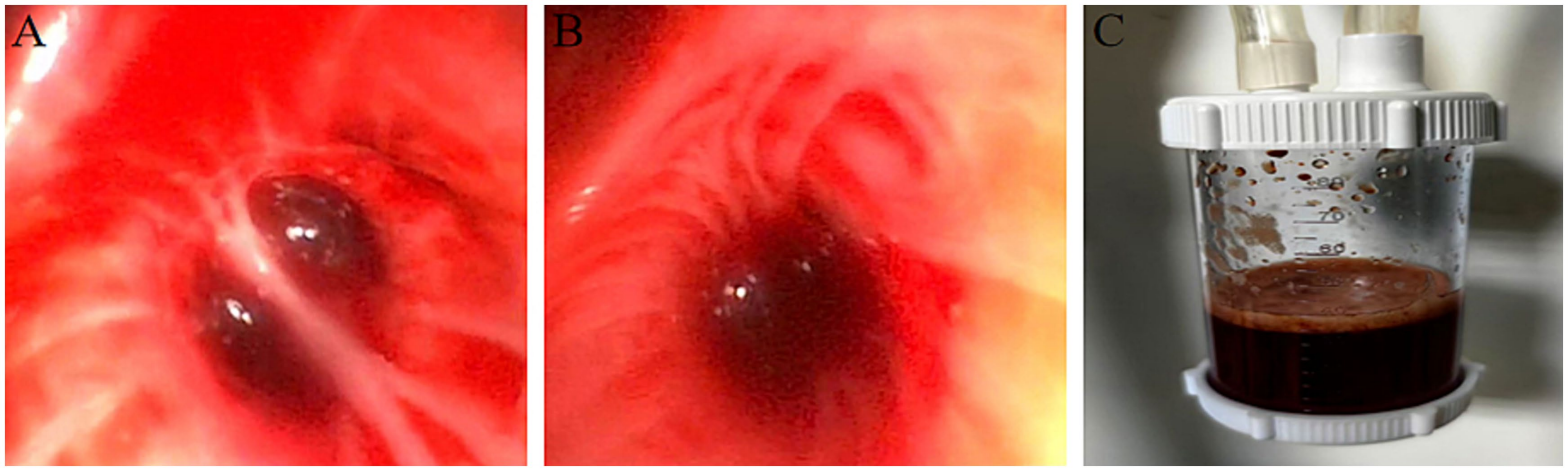
Bronchoscopic photos. **(A–C)** Copious blood-tinged fluid within the tracheal lumen.

A 35-year-old male with an more than 10-min drowning-induced cardiorespiratory arrest presented comatose. Physical examination revealed bulbar conjunctival edema with petechial hemorrhages, sluggish pupillary light reflexes (bilateral), and abdominal distension. Vital signs included weight 60 kg, temperature 36°C, heart rate 125 bpm, and blood pressure 114/56 mmHg under norepinephrine infusion (0.2 μg/kg/min). Mechanical ventilation was ongoing, with a functional right-sided closed thoracostomy tube draining copious air (chest drainage system). Laboratory studies demonstrated hepatic dysfunction, acute kidney injury, and coagulopathy (Table 1). Bronchoscopy identified abundant bloody secretions in the bronchial tree (Figures 2A–C). No significant preexisting comorbidities were reported by the family. Initial diagnoses included acute respiratory distress syndrome (ARDS), pneumothorax, and refractory hypoxemia.

**Table 1:** Laboratory examinations.

Variable	Reference range, this hospital^a^	Admission	ECMO 72 h	Discharge
Blood routine examination				
White blood cell (×10^9^/L)	3.50–9.50	22.83	15.98	4.53
Red blood cell (×10^12^/L)	4.3–5.8	3.71	3.21	3.11
Hemoglobin (g/L)	130–175	109	94	89
Platelet (×10^9^/L)	125–350	115	79	193
Absolute neutrophil count (×10^9^/L)	1.80–6.30	21.64	14.76	2.98
Absolute lymphocyte count (×10^9^/L)	1.1–3.2	0.5	0.64	1.08
Renal function				
Blood urea (mmol/L)	3.1–8.0	10.6	9.9	7.9
Serum creatinine (μmol/L)	57–97	170	99	84
Glomerular filtration rate (mol/min)	80–120	44.1	84.8	103.4
Uric acid (μmol/L)	202–463	605	192	247
Liver function				
Alanine aminotransferase (U/L)	9.0–50.0	318.8	64.6	41.8
Aspartate aminotransferase (U/L)	15–40	293.8	36.2	32
Total protein (g/L)	65.0–85.0	53.1	63.4	58.6
Albumin (g/L)	40.0–55.0	37.8	43.0	36.8
Globulin (g/L)	20.0–40.0	15.3	21.6	21.8
Coagulation function				
Prothrombin time activity (%)	70.0–130.0	50.5	64.1	85.5
Thrombin time (s)	14–21	35.1	14.8	15.9
Activated partial thromboplastin time (s)	23.3–32.5	89.8	28.2	27.9
Fibrinogen (g/L)	1.8–3.5	1.26	3.08	3.74
D-Dimer (mg/mL)	<0.55	>80	7.48	6.21
Other				
C-reactive protein (mg/L)	0–8	30	16.4	2
Procalcitonin (ng/mL)	<0.046	19.2	0.1	0.035

Serial monitoring of blood parameters showed initial abnormalities on admission and progressive normalisation by discharge.

a Reference values are influenced by many variables, including the patient population and the laboratory methods used.

**Figure 3:**
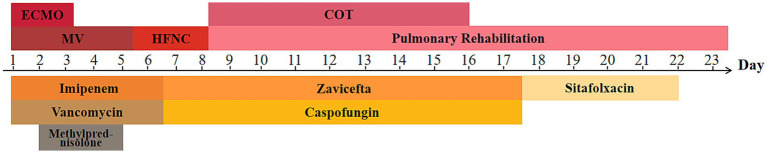
Therapeutic management flow chart. The upper panel delineates the chronological sequence of ECMO combined with stepwise oxygen therapy weaning, while the lower panel documents temporal adjustments in the antimicrobial stewardship regimen.

Our therapeutic interventions are as follows:

- *Respiratory & hemodynamic support*: Maintained closed thoracostomy drainage with analgesia-sedation; initiated double-lumen nasogastric tube insertion for gastrointestinal decompression.
- *Airway management*: Performed therapeutic bronchoscopy, aspirating hemorrhagic secretions.
- *Coagulopathy correction*: Administered fresh frozen plasma to restore coagulation homeostasis.
- *ECMO initiation*: Despite 6 h of lung-protective mechanical ventilation (PEEP 12 cmH₂O, FiO₂ 100%), refractory hypoxemia persisted (PaO₂/FiO₂ ratio <80), prompting veno-venous ECMO (V-V ECMO) cannulation.
- *Adjunctive therapies:* Implemented prone positioning (12 h/day) to enhance pulmonary recruitment; initiated targeted temperature management (36°C for 72 h) to mitigate hypoxic–ischemic brain injury.
- *Antimicrobial stewardship*: Empirical meropenem combined with vancomycin was administered on admission. Fever (38.4°C) emerged on day 6, with bronchoalveolar lavage cultures and metagenomic next-generation sequencing (mNGS) identifying *Pseudomonas aeruginosa*, *Klebsiella pneumoniae*, and *Candida glabrata*. Antifungal therapy (caspofungin) and zavicefta were subsequently escalated (Figure 3). On day 17 sitafolxacin was de-escalated.
- *Supportive care*: Enforced strict infection control protocols, normothermia maintenance, and enteral nutrition optimization via a standardized ICU bundle.

**Figure 4:**
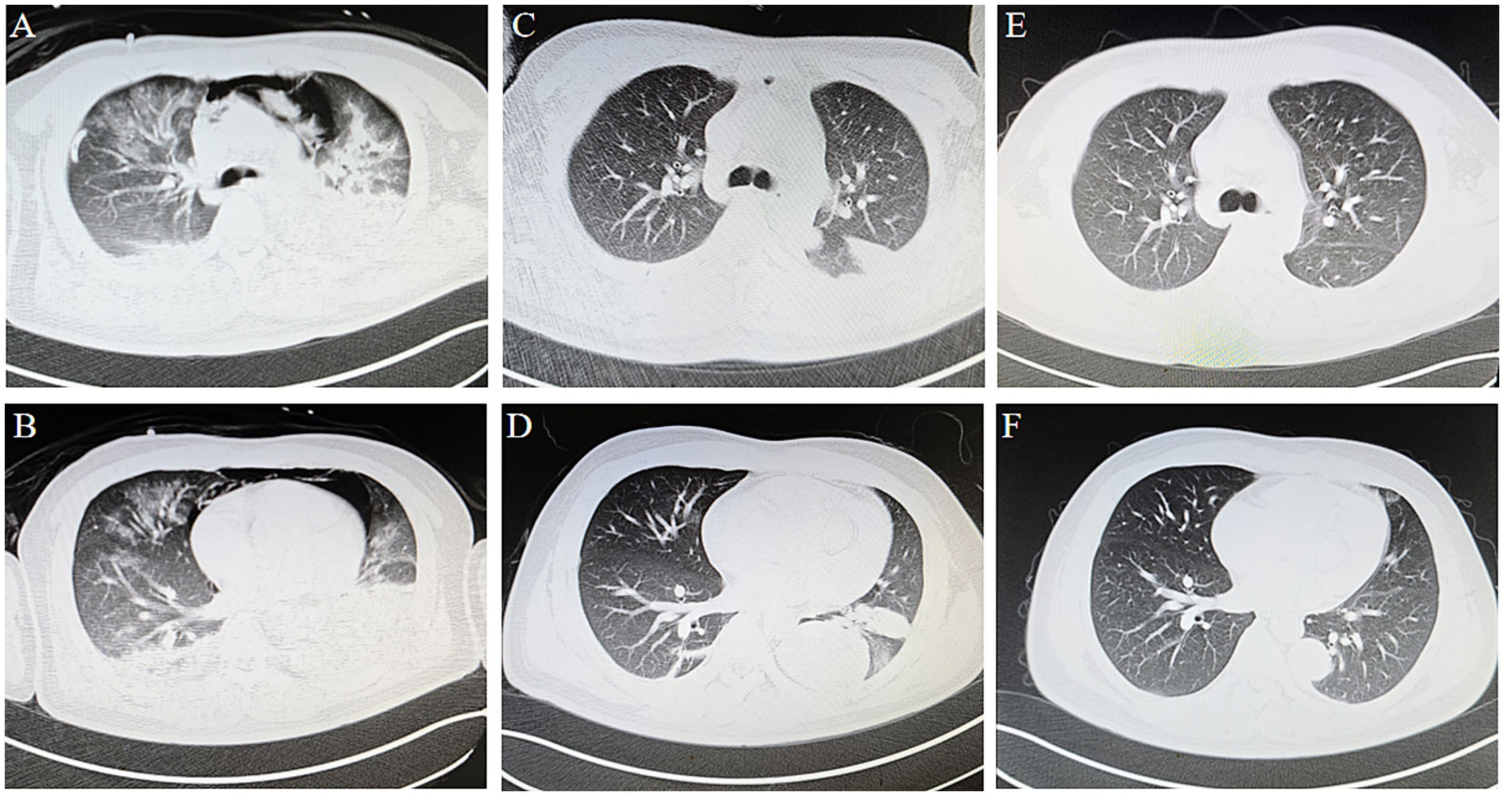
Thoracic CT. **(A,B)** At 72 h of ECMO, CT demonstrating bilateral pulmonary edema with extensive ground-glass opacities, patchy consolidations and pneumomediastinum. **(C,D)** Thoracic imaging demonstrated complete resolution of pulmonary edema, with residual consolidative opacities persisting in bilateral lower lobes. **(E,F)** The 3-month follow-up showed that the patchy shadows were significantly reduced, and had basically recovered.

As the patient’s oxygenation improved, ECMO parameters were progressively weaned while ventilator support was concomitantly increased. By 72 h post-ECMO initiation, serial chest radiographs demonstrated marked resolution of the right pneumothorax (Figure 1B). Thoracic CT revealed persistent diffuse bilateral pulmonary edema with extensive ground-glass opacities and consolidative patches (Figures 4A,B). Concurrently, laboratory indices showed progressive normalization of hepatic and renal function (Table 1). Subsequently, the patient was successfully weaned from ECMO.

Post-decannulation respiratory management included mechanical ventilation coupled with scheduled bronchoscopic airway clearance. During the 48-h post-decannulation period, the patient passed spontaneous breathing trials (SBTs) and cuff-leak tests, facilitating extubation. Oxygenation support was then transitioned to high-flow nasal cannula (Supplementary material) to promote alveolar recruitment, complemented by a structured pulmonary rehabilitation program. Sequential stepwise de-escalation to conventional nasal oxygen (2–3 L/min) was achieved by day 8, with complete oxygen independence documented on hospital day 16 (see Figure 3 for timeline). Parameter settings for ECMO, MV, etc. during salvage are shown in the Supplementary material.

By ICU day 23, follow-up thoracic CT demonstrated substantial pulmonary recovery with residual left lower lobe consolidative opacities (Figures 4C,D), paralleled by normalization of hepatic and renal function biomarkers (Table 1). These improvements prompted ICU discharge to general ward care. No ECMO-or ventilator-associated complications occurred during hospitalization.

At the 3-month outpatient follow-up, repeat imaging revealed near-complete resolution of consolidative lesions (Figures 4E,F). The patient reported full resumption of premorbid activities (6-min walk distance: 420 meters) without exertional dyspnea (mMRC grade 0). At the 9-month follow-up, the patient reported that tests at other hospitals showed that the indicators had returned to normal.

## Discussion

Drowning as a critical global public health challenge, underscored by the United Nations’ inaugural World Drowning Prevention Day (July 2021) and a 38% decline in age-standardized drowning mortality since 2000. While these advances reflect progress in prevention strategies, achieving the SDG target (reducing unintentional injury deaths) demands accelerated multisectoral collaboration (3). Concurrently, optimizing resuscitation protocols for salvageable patients remains imperative.

This case exemplifies the intricate balance between aggressive intervention and iatrogenic risk mitigation in drowning-induced ARDS. Despite early lung-protective ventilation, refractory hypoxemia (PaO₂/FiO₂ <80) and barotrauma-related pneumothorax necessitated escalation to V-V ECMO—a decision aligned with recent ELSO guidelines prioritizing early ECMO for reversible respiratory failure (8). Our weaning protocol, characterized by synchronized ECMO flow reduction and incremental ventilator adjustments, achieved decannulation within 72 h—a timeframe associated with lower nosocomial infection risks compared to prolonged ECMO runs (12). Post-ECMO respiratory management leveraged sequential oxygen therapy (HFNC → NIV → conventional oxygen), which may attenuate reintubation rates (13).

Notably, the delayed febrile response (day 6) underscores drowning’s unique infection dynamics. Unlike typical aspiration pneumonitis, water immersion predisposes to polymicrobial infections with pathogens (14). Our antimicrobial approach—empirical treatment followed by culture-guided escalation—de-escalation. We believe that an appropriate antimicrobial stewardship strategy is also important, and therefore antibiotics were actively used in the management of this patient.

This case demonstrates ECMO’s viability as rescue therapy and offers valuable insights for analogous clinical scenarios. Yet three evidence gaps warrant prioritization: optimal ECMO initiation thresholds (EOLIA criteria vs. drowning-specific indices); standardized weaning biomarkers beyond PaO₂/FiO₂ (e.g., ventilatory ratio, dynamic compliance); cost-effectiveness of sequential oxygen modalities in resource-constrained settings. Prospective RCTs comparing ECMO timing strategies (EOLIA vs. RESCUE trials) and bundled rehabilitation protocols are urgently needed to establish drowning-specific care pathways.

## Data Availability

The original contributions presented in the study are included in the article/Supplementary material, further inquiries can be directed to the corresponding author.
